# Salt stress perception and metabolic regulation network analysis of a marine probiotic *Meyerozyma guilliermondii* GXDK6

**DOI:** 10.3389/fmicb.2023.1193352

**Published:** 2023-07-17

**Authors:** Xinghua Cai, Huijie Sun, Bing Yan, Huashan Bai, Xing Zhou, Peihong Shen, Chengjian Jiang

**Affiliations:** ^1^State Key Laboratory for Conservation and Utilization of Subtropical Agro-Bioresources, Guangxi Research Center for Microbial and Enzyme Engineering Technology, College of Life Science and Technology, Guangxi University, Nanning, China; ^2^Guangxi Key Lab of Mangrove Conservation and Utilization, Guangxi Mangrove Research Center, Guangxi Academy of Sciences, Beihai, China; ^3^National Engineering Research Center for Non-Food Biorefinery, State Key Laboratory of Non-Food Biomass and Enzyme Technology, Guangxi Research Center for Biological Science and Technology, Guangxi Academy of Sciences, Nanning, China

**Keywords:** *Meyerozyma guilliermondii*, salt stress perception, metabolic regulation 3 network, integrative omics technology, functional products

## Abstract

**Introduction:**

Extremely salt-tolerant microorganisms play an important role in the development of functional metabolites or drug molecules.

**Methods:**

In this work, the salt stress perception and metabolic regulation network of a marine probiotic *Meyerozyma guilliermondii* GXDK6 were investigated using integrative omics technology.

**Results:**

Results indicated that GXDK6 could accept the salt stress signals from signal transduction proteins (e.g., phosphorelay intermediate protein YPD1), thereby contributing to regulating the differential expression of its relevant genes (e.g., *CTT1*, *SOD*) and proteins (e.g., catalase, superoxide dismutase) in response to salt stress, and increasing the salt-tolerant viability of GXDK6. Omics data also suggested that the transcription (e.g., *SMD2*), translation (e.g., *MRPL1*), and protein synthesis and processing (e.g., inner membrane protein OXA1) of upregulated RNAs may contribute to increasing the salt-tolerant survivability of GXDK6 by improving protein transport activity (e.g., Small nuclear ribonucleoprotein Sm D2), anti-apoptotic ability (e.g., 54S ribosomal protein L1), and antioxidant activity (e.g., superoxide dismutase). Moreover, up to 65.9% of the differentially expressed genes/proteins could stimulate GXDK6 to biosynthesize many salt tolerant-related metabolites (e.g., β-alanine, D-mannose) and drug molecules (e.g., deoxyspergualin, calcitriol), and were involved in the metabolic regulation of GXDK6 under high NaCl stress.

**Discussion:**

This study provided new insights into the exploration of novel functional products and/or drugs from extremely salt-tolerant microorganisms.

**Graphical Abstract:**
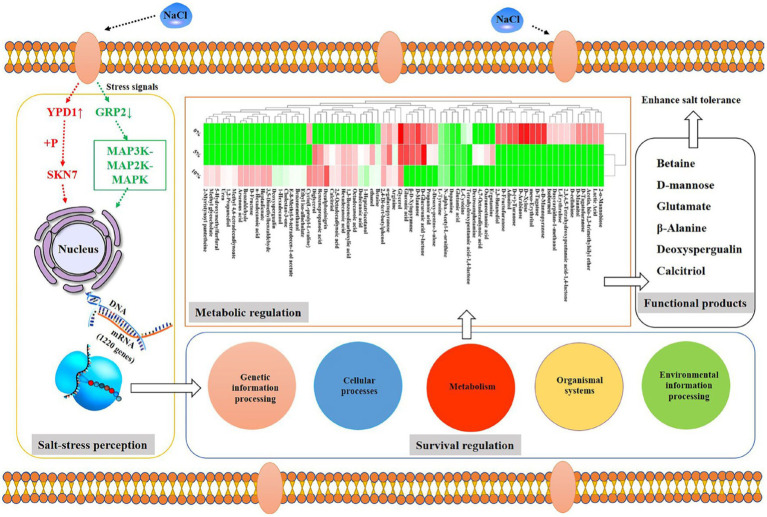

## Introduction

Biosynthesis of new functional metabolites by extremely salt-tolerant probiotics has become a research hotspot in the field of synthetic biology (Li et al., 2021). The unique metabolic regulation mechanisms of extremely salt-tolerant probiotics support their survival under high salt stress, which is of great significance for developing new functional metabolites (Xu et al., 2014). However, the regulatory mechanism of how salt-tolerant probiotics survive under salt stress and produce functional metabolites is still unclear. Typical salt-intolerant probiotics, including *Lactobacillus, Saccharomyces cerevisiae*, and *Bifidobacterium*, cannot grow when the salt concentration is increased by more than 8% NaCl (Palomino et al., 2010; Park et al., 2015; Piqué et al., 2019). However, salt-tolerant probiotics have shown great potential for development. For instance, they could survive well under salt stress and regulate their unique survival mechanism to resist or adapt to high salt stress, and it seems that they are more likely to produce abundant functional metabolites under stress conditions (Silva et al., 2014). Therefore, screening extremely salt-tolerant probiotics (≥10% NaCl) and revealing their salt tolerance survival and metabolic networks have become more interesting than before (Serra et al., 2019; Banciu et al., 2020).

In order to cope with salt stress, studies show that probiotics have evolved various strategies, including intracellular microenvironment, cell membranes, and protein synthesis, to resist salt injury (Matsushika et al., 2017). However, few reports focused on how they survive under salt stress and produce functional metabolites. Yang et al. (2019) introduced a natural salt-resistant *Meyerozyma guilliermondii* isolated from mangrove ecosystems, and studied its salt-resistant mechanism by comparative transcriptomics. They found that many annotated salt-tolerant genes contributed to the survival of *M. guilliermondii* (e.g., *FPS1*, *GPD1*). Similar results were also reported by Hou et al. (2013), who revealed the salt tolerance mechanisms of *Zygosaccharomyces rouxii* by comparative physiological and transcriptomic analyses, and found that salt stress induced the accumulation of glycerol and trehalose and an increase of the proportion of unsaturated fatty acids. In addition, the genes involved in cellular metabolism and ribosome biosynthesis were expressed differentially. However, few reports reported the global effect of salt stress on the survival regulation network of salt-tolerant yeast (Ghaemmaghami et al., 2003). The relationship between key genes, proteins, and metabolites relevant to salt tolerance remains unknown. Furthermore, few reports focused on the influence of functional metabolites on the salt tolerance survival of *M. guilliermondii* (Eichner et al., 2014). Thus, exploring how salt-tolerant genes regulate the expression of salt tolerance-related proteins, what aspects of cell functions will be affected by these proteins, and whether they could produce relevant metabolites that are conducive to supporting the salt tolerance survival of *M. guilliermondii* or not are necessary. The solutions to these bottlenecks could contribute to revealing survival and metabolic networks of probiotics under high salt stress (Bi et al., 2015; Peinemann et al., 2020). They are also conducive to the development of novel functional products and/or drugs from extremely salt-tolerant microorganisms.

A previous study has provided a marine multi-tolerant *M. guilliermondii* GXDK6, which showed high salt resistance (up to 12% NaCl or 18% KCl; Mo et al., 2021). Because of its remarkable salt-tolerant survivability, GXDK6 was hypothesized to survive well under high salt stress by regulating its related genes to control the expression of key salt-tolerant proteins, which may contribute to regulating its cell function and/or producing some beneficial functional metabolites. To test this hypothesis, integrative omics technology was used to investigate the salt stress perception and metabolic regulation network in GXDK6. This work is helpful to understand the metabolic regulation in GXDK6 and developing some functional metabolites or drugs from extremely salt-tolerant microorganisms.

## Materials and methods

### Species and reagents

*Meyerozyma guilliermondii* GXDK6 was obtained from the subtropical mangrove sediments in the Beibu Gulf of the South China Sea (21°29′25.74′′ N, 109°45′49.43′′ E). It has been deposited in the China General Microbiological Culture Collection Center (CGMCC) under CGMCC no. 16007. N-methyl-trimethyl-silyl-trifluoroacetamide (MSTFA) and methoxyamine pyridine hydrochloride were of chromatographic grade and purchased from Sigma–Aldrich, Inc. (Darmstadt, Germany). Glucose, yeast powder, agar powder, peptone, HCl, NaOH, and NaCl were of analytical grade and were purchased from Novagen (Darmstadt, Germany). The universal total RNA rapid extraction kit, the YfxScript 1st strand cDNA Synthesis Kit, and the 2XSYBR Green Fast qPCR Master Mix kit were purchased from Yifeixue Biotechnology Co., Ltd. (Nanjing, China).

### Physicochemical characteristics and salt tolerance of GXDK6

GXDK6 was incubated at 37°C and 200 rpm for 24–48 h in YPD medium containing 0, 5, 10, 12, 15, and 18% NaCl, respectively. Three replicates were set for each condition. The growth of GXDK 6 under different NaCl stresses was evaluated by turbidimetric detection. The strains were collected by centrifugation at 8,000 × *g* for 10 min, and then observed by scanning electron microscope (Carl Zeiss AG, Oberkochen, Germany). According to the growth of GXDK 6 under different salt stresses, the NaCl concentration range suitable for GXDK 6 stress was screened out to prepare for subsequent experiments. In addition, a salinometer (Shanghai Speedway Biotechnology Co., Ltd., Shanghai, China) was used to detect the salt concentration in the fermentation broth to evaluate the salt-removal ability of GXDK6 (Shin and Lee, 2014).

### Genome sequencing analysis of GXDK6

The genomic DNA of GXDK6 was extracted according to the reported methods with slight modifications (Andreou, 2013). The purity of the extracted DNA was detected by PCR and agarose gel electrophoresis (Jamasbi et al., 2004). The ITS gene was amplified by PCR using ITS1 (5′-TCCGTAGGTGAACCTGCGG-3′)/ITS4 (5′-TCCTCCGCTTATTGATATGC-3′) universal primers (Dupont et al., 2019). The ITS sequencing data of GXDK6 were deposited to the National Microbiology Data Center database^1^ under the accession number NMDCN000022O. The whole-genome sequencing data of GXDK6 were deposited in the National Microbiology Data Center database (see footnote 1) under accession number NMDC60014229 (Mo et al., 2021).

### Transcriptome sequencing analysis of GXDK6

GXDK6 strain was cultured at 0, 5, and 10% NaCl for 16 h at 37°C and 200 rpm, respectively. Three biological replicates were set for each condition. Subsequently, the cells were collected by centrifuging at 8,000 × *g* for 10 min and then transferred into liquid nitrogen for rapid freezing. The total RNA in GXDK6 was extracted using the trizol method (Villa-Rodríguez et al., 2018). The mRNA was enriched by oligo (dT) beads and purified by removing rRNA using a Ribo-ZeroTM Magnetic Kit (Epicenter, Madison, WI, United States). The mRNA quality was assessed on an Agilent 2100 Bioanalyzer (Agilent Technologies, Palo Alto, CA, United States) and was checked using RNase-free agarose gel electrophoresis. The enriched mRNA was fragmented into short fragments by using fragmentation buffer (100 mM Tris-EDTA) and reverse transcripted into cDNA with random primers. Second-strand cDNA was synthesized by DNA polymerase I, RNase H, dNTPs, and a Tris-EDTA buffer (100 mM Tris–HCl, 10 mM EDTA). Then, the cDNA fragments were purified with a QiaQuick PCR extraction kit (Qiagen, Venlo, The Netherlands), end repaired, poly(A) added, and ligated to Illumina sequencing adapters. The ligation products were size selected by agarose gel electrophoresis, PCR amplified, and sequenced using Illumina HiSeq2500 by Gene Denovo Biotechnology Co., Ltd. (Guangzhou, China). The differential expression analysis of the RNAs was performed on DESeq2 software. The genes/transcripts with false discovery rate below 0.05 and fold change ≥2 were considered the DEGs/transcripts.

### Proteomics analysis of GXDK6

GXDK6 was incubated for 16 h under 0, 5, and 10% NaCl stress, respectively. The cells were collected by centrifugation at 8,000 × *g* for 10 min. The total proteins in GXDK6 were extracted by SDT lysis methods (Zhu et al., 2014). Proteome sequencing and analysis of the extracted proteins were conducted using a tandem mass tag (TMT)-based quantitative proteomics (Myers et al., 2018). TMT-labeled peptides were fractionated by RP chromatography using Agilent 1260 infinity II HPLC. LC–MS/MS analysis was performed on a Q Exactive plus mass spectrometer (Thermo Fisher Scientific) that was coupled to Easy nLC (Thermo Fisher Scientific) for 60/90 min. The MS/MS raw files were processed using MASCOT engine (Matrix Science, London, United Kingdom; version 2.6) embedded into Proteome Discoverer 2.2, and searched against the NCBI/NR/UniProt database. Proteins with fold change>1.2 and *p* value (Student’s *t* test) <0.05 were considered as differentially expressed proteins (DEPs).

### Metabolomics analysis of GXDK6

GXDK6 cells were removed by centrifugation at 12,000 × *g* for 10 min after incubation for 16 h. The zymotic fluid was then filtered by a 0.45 μm microporous membrane (organic system), and placed in a 1.5 mL centrifuge tube. Subsequently, 500 μL of the above fermentation broth from each condition was transferred into new 1.5 mL centrifuge tubes, and 5 μL of ribitol solution (a special reagent for GC analysis) was added in the tubes as the internal standard at a concentration of 1 mg/mL. Afterward, all the samples were concentrated in a freeze centrifugal concentrator until they were completely dried into powder or flocculent. Then, they were used for gas chromatography–mass spectrometry (GC–MS) detection and analysis.

All the samples required a two-step pretreatment before performing GC–MS detection. First, the alkylation reaction of the samples was performed by adding 80 μL methoxyamine pyridine hydrochloride solution (a special reagent for GC analysis) with a concentration of 20 mg/mL and then oscillating in a rotary shaker at 200 rpm and 37°C for 120 min. Second, when the alkylation reaction was completed, 80 μL of MSTFA was added to the samples for derivatization reaction, which was also conducted in a rotary shaker at 200 rpm and 37°C for 120 min. Subsequently, the samples were centrifuged at 12,000 × *g* for 10 min to collect the supernatant for GC–MS detection.

The above samples were detected using DSQ II single quadrupole GC–MS (Thermo Fisher Company). The injection port temperature of the instrument was maintained at 250°C. One μL of the derivative sample was extracted and injected into the dodecyl benzene sulfonic acid column (length: 30 mm × inner diameter: 250 μm × thickness: 0.25 μm). The source temperature of the mass spectrometer was maintained at 250°C under the direct ionization mode of 70 eV ionization energy and 8,000 V accelerating voltage. In the full scan and selective ion recording experiments, the quadrupole temperature was maintained at 150°C. The initial gas chromatograph temperature was set at 85°C for 5 min and then increased to 330°C at a rate of 15°C/min. Helium was used as the carrier gas with a constant flow rate of 1 mL/min, and the operating range of mass spectrometry was 50–600 M/Z (Li et al., 2016).

### RT-qPCR verification

Three significantly upregulated genes (*STL1, CWP1,* and *CAR1*) and three significantly downregulated genes (*INO1, GRP2,* and *TKPR1*) were selected for RT-qPCR verification (these genes were located in the third quadrant or seventh quadrant, indicating that the mRNAs and proteins showed the same differential expression trend, and that they may have a more stable regulation process under salt stress). The size of primers and product are shown in Table 1. The total RNA in GXDK6 was extracted using the universal total RNA rapid extraction kit (Yifeixue Biotechnology Co., Ltd., Nanjing, China). First-strand cDNA was synthesized using the YfxScript 1st strand cDNA Synthesis Kit. RT-qPCR reaction was executed at the final concentrations of 20–25 ng/μL template DNA, 250 ng/μL of forward primer, 250 ng/μL of reverse primer, and adding appropriate 2xSYBR Green Fast qPCR Master Mixture/−LR/HR and RNase-free H_2_O. The mixture was heated to 95°C for 2 min, followed by 40 cycles of denaturation at 95°C for 15 s, anneal/extension at 60°C for 30 s, and then increased by 0.3°C/10 s from 63 to 95°C to obtain the melting curve. The samples were reacted with a high-throughput real-time fluorescence quantitative PCR. Meanwhile, fluorescence detection was performed on a LightCycler 480 Software Release 1.5.1.62 SP3. The relative quantitation of the selected genes was calculated using the 2-ΔΔCt method (Livak and Schmittgen, 2001).

**Table 1:** Selected genes in GXDK6 and their specific primers for RT-qPCR.

Genes	Gene ID	Primer	Product size (bp)
*STL1*	scaffold4.g376	F:ATTGGATGTTTTTTCGGTGC R:GTTCGTGATTTAATGTCCCAAACCA	215
*CWP1*	scaffold1.g788	F:TCCTCTTCCGTTTTGGTCTA R:TGCTTGTTGTTTAAHTGSSACHTGS	191
*CAR1*	scaffold3.g109	5-GAAGACAAGAAGACCGATGA-3 3-TGTTTTTTTCACTTACTAGAATCCA-5	214
*INO1*	scaffold3.g246	F: ACCAGTAACTCAAAAGCGTC R: TGAGATTCGAGGAGAGATTGCAGTA	115
*GRP2*	scaffold3.g827	F: CAACAATCTTTGACCAGCAG R: TTAAGCAATATGTTGATAACAATTA	185
*TKPR1*	scaffold5.g49	5-AAAAGGACCAACTAACCATACC-3 3-ACGAAAAGCTGTTACCTGTGAAATA-5	146
*ACT1*	Reference gene	F: CTTACGAGTTGCCCGATGGT R: CAAGTCGGAAGGACGGAACA	86

### Effect of adding exogenous metabolites on the growth of GXDK6

GXDK6 was incubated at 37°C and 200 rpm for 48 h in YPD media containing 10% (w/v) NaCl as the control. At the same time, the final concentration of β-alanine, betaine, D-mannose, urea, and L-cysteine was 100 mg/L in different experimental groups, and the incubation conditions were the same as those of the control group. The number of viable fungi was detected by a plate coating.

### Cloning of *YPD1* gene from *Meyerozyma guilliermondii* GXDK6 in *Escherichia coli*

According to the genome data of GXDK6, the length of *YPD1* gene is 458 bp. In this work, it was completely synthesized by artificial chemical synthesis. The cloning vector selected for gene expression was ppic9k with kanamycin resistance. The restriction site was EcoRI/NotI. The protocol of cloning *YPD1* gene in *E. coli* was according to the method of Gohel and Singh (2012) and slightly modified. The positive clones were screened by kanamycin resistance test and agarose gel electrophoresis verification. Sanger sequencing method (Daniels et al., 2021) was used to verify whether the inserted sequence was consistent with the reference sequence. After the cloned strains were obtained, they were cultured in LB medium containing 1–10% NaCl for 48 h, and the salt-tolerant viability of the cloned strain was detected and evaluated by plate counting method.

### Data analysis

Data fitting and mapping analysis were performed using Origin 2018. Statistical analysis of the other experimental data was performed using SPSS 17.0, and *p* value <0.05 indicated significant differences. For the transcriptome and proteome data, the differential genes and proteins were annotated using KEGG and GO databases. For the data obtained by GC–MS, Xcalibur software was used to extract the peak area of the total ion chromatogram. The differential metabolites were analyzed on GraphPad Prism version 5.01 (La Jolla, CA, United States), and the heatmap analysis was performed on R software.

## Results

### Tolerance of GXDK6 to salt stress

**Figure 1:**
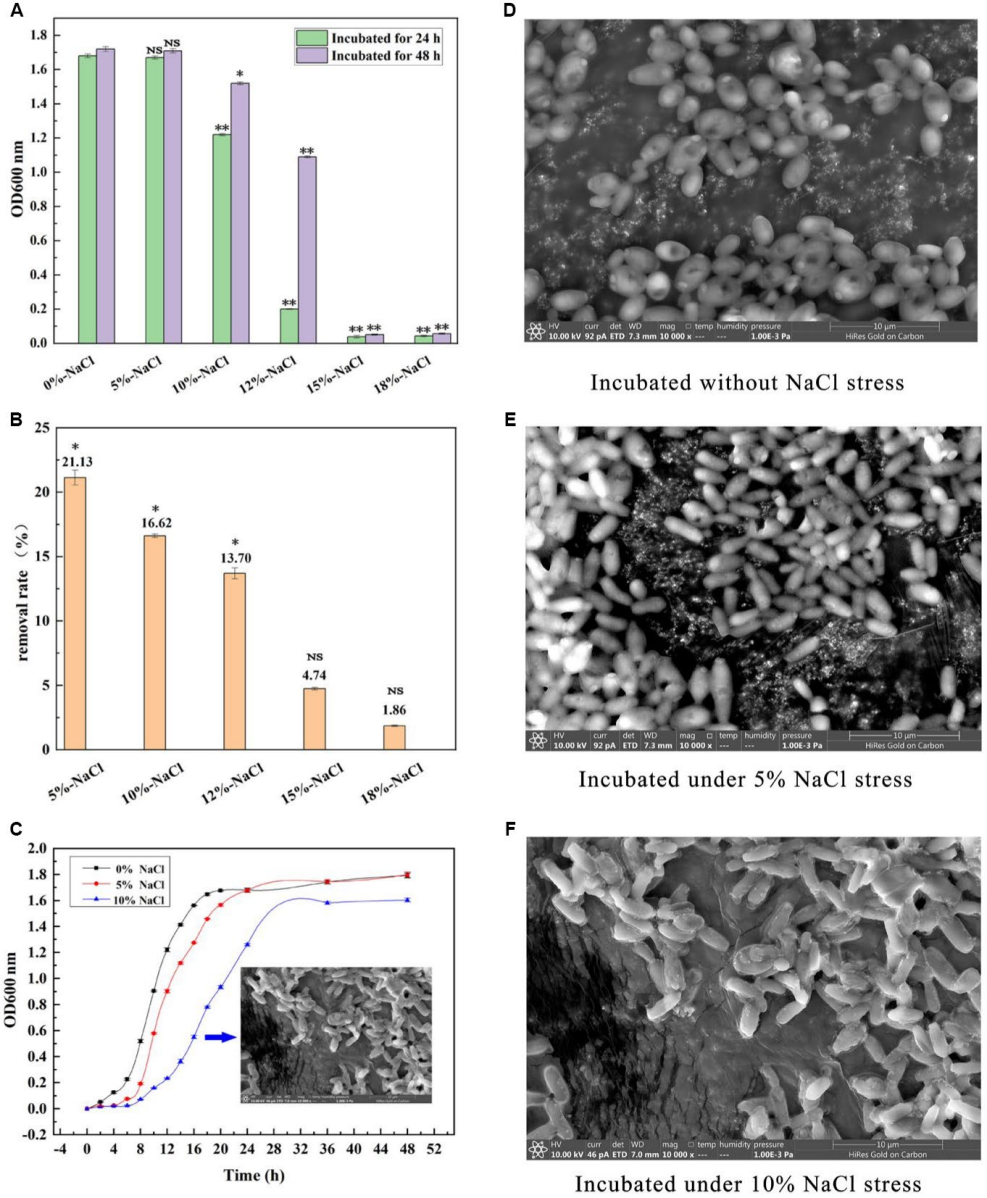
Salt tolerance of GXDK6. **(A)** Incubation of GXDK6 under 0–18% NaCl; **(B)** Salt removal rate of GXDK6 cultured for 48 h; **(C)** Growth curve of GXDK6 incubated under 0–10% NaCl. **(D)** Cell morphology of GXDK6 incubated without NaCl stress; **(E)** Cell morphology of GXDK6 incubated under 5% NaCl stress; **(F)** Cell morphology of GXDK6 incubated under 10% NaCl stress. “^**^” indicated very significant difference compared with the control (*p* < 0.01); “^*^” indicated significant difference compared with the control (*p* < 0.05)”; and “NS” indicated not significant difference compared with the control (*p* > 0.05).

As shown in Figure 1A, GXDK6 grew well within 10% NaCl stress. On the contrary, when the concentration of NaCl increased to 12%, it was obviously inhibited and could hardly grow under the condition of 15% NaCl. Moreover, GXDK6 demonstrated a great salt-removal ability. Up to 21.1% NaCl could be removed by GXDK6 after being incubated for 48 h under 5% NaCl stress. With the increase of NaCl concentration, the salt removal rate gradually decreased (Figure 1B). In order to further understand the growth regulation of GXDK6 under salt stress, it was incubated under 0, 5, and 10% NaCl stress, respectively. Results showed that when the NaCl stress increased from 0 to 10%, the growth of GXDK 6 in the lag phase was prolonged and the logarithmic phase was inhibited (Figure 1C), which indicated that high NaCl stress may interfere with DNA replication and protein synthesis in GXDK6. Moreover, the surface morphology of GXDK 6 remained round or oval when there is no NaCl stress (Figure 1D), and gradually shrinks or stretches under 5–10% NaCl stress (Figures 1E,F). These findings suggest that GXDK6 may need more time to regulate the expression of its cell membrane and/or cell wall-related genes/proteins, transform its cell metabolism, and increase the internal osmotic pressure of cell to adapt to high salt stress.

### Omics analysis of GXDK6 under salt stress

**Figure 2:**
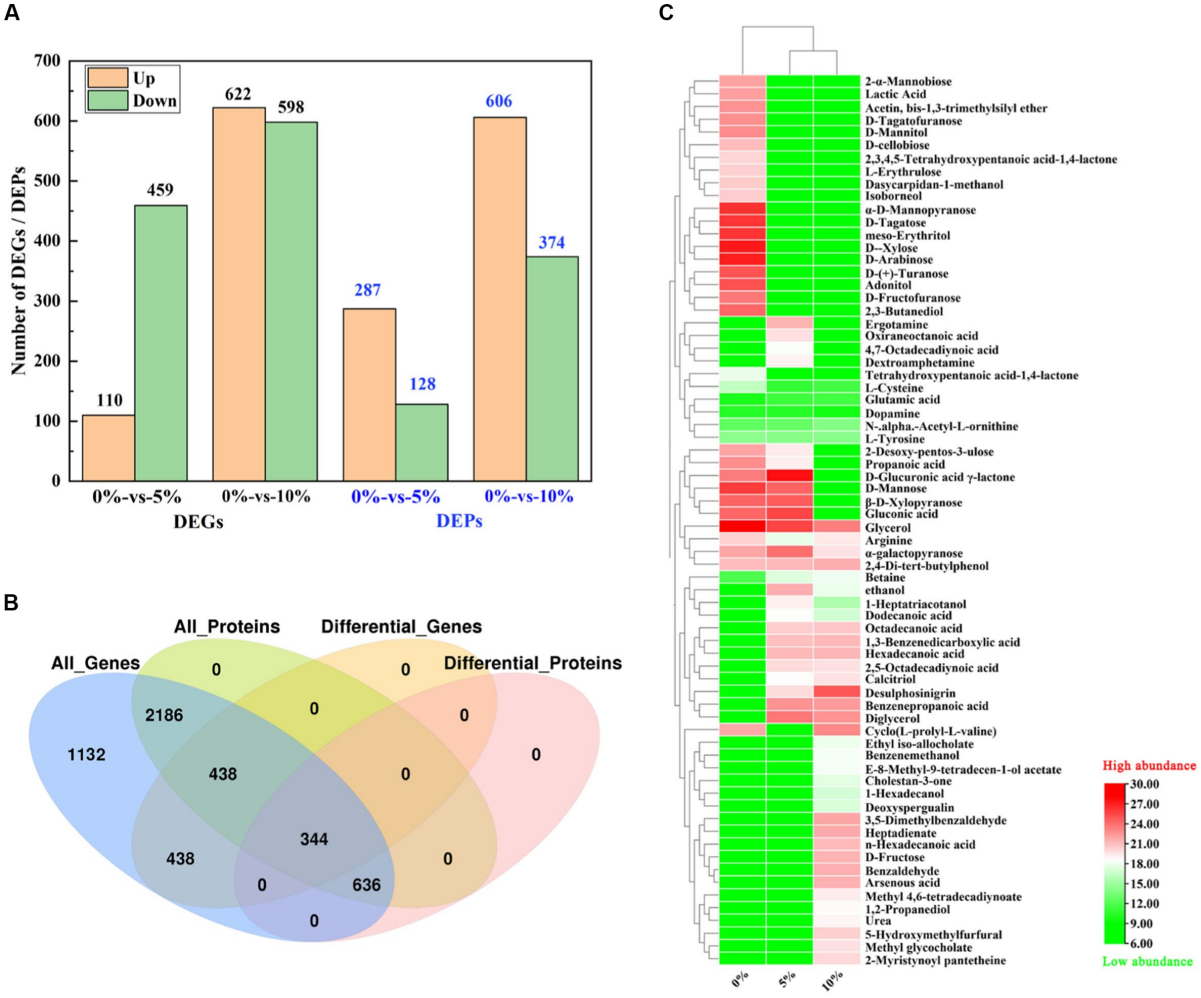
Integrative omics analysis of GXDK6 under high NaCl stress for 16 h. **(A)** Number of DEGs and DEPs in GXDK6 under 0–10% NaCl stress; **(B)** Venn diagram analysis of DEGs and DEPs; **(C)** Metabolomics analysis of GXDK6 under NaCl stress.

According to the whole-genome data of GXDK6, 307 genes in GXDK6 (Supplementary Table S1) were annotated as the same genes referenced to the known *Saccharomyces* Genome Database (SGD; https://www.yeastgenome.org/observable/APO:0000204#annotations). However, the RNA-sequencing analysis of GXDK6 identified 1,220 genes that were significantly upregulated or downregulated (fold change ≥ 2.0, *p* < 0.05) under 10% NaCl stress (Supplementary Table S2). Among the 1,220 DEGs (accounting for 23.57% of 5,175 reference genes), 622 were upregulated and 598 were downregulated (Figure 2A). These DEGs were linked to five life activities of GXDK6 (genetic information processing, cellular processes, metabolism, organismal systems, and environmental information processing) and especially involved in the metabolic regulation of GXDK6. The results of RT-qPCR also confirmed some important regulatory genes (e.g., *STL1, CWP1*) were differentially expressed under NaCl stress (Supplementary Figure S1). In the past, the research on the salt tolerance mechanisms of yeast usually began with the identification and annotation analysis of the genes, and most of the research took SGD as a reference database. However, natural *S. cerevisiae* is not an extremely salt tolerance microorganism (its salt tolerance is often within 8% NaCl), suggesting that SGD is incomplete when used for extremely salt tolerance *M. guilliermondii*. Therefore, this work provides an important reference for further study on the salt tolerance mechanism of other extremely salt-tolerant microorganisms.

Proteomics analysis demonstrated that 3,604 proteins (Supplementary Table S3) were detected in GXDK6 under 5–10% NaCl stress, among which, 287 differentially expressed proteins (DEPs) were upregulated and 128 DEPs were down-regulated under 5% NaCl stress for 16 h (Supplementary Table S4), accounting for 11.51% of the total proteins detected. However, the number of DEPs increased to 980 (Figure 2A; Supplementary Table S5) when GXDK6 was stressed under 10% NaCl for 16 h (606 upregulated and 374 downregulated proteins), accounting for 27.19% of the total proteins detected. Importantly, all the DEPs matched well with their corresponding DEGs based on the Kyoto Encyclopedia of Genes and Genomes (KEGG) and Gene Ontology (GO) annotation analysis (Figure 2B; Supplementary Table S6), suggesting that the regulatory expressions of DEGs and DEPs have great correlation. According to the transcriptomics and proteomics analysis results, these DEGs/DEPs were involved in transforming the metabolic network of GXDK6, which may contribute to producing some differential metabolites that support the viability of GXDK6 under NaCl stress.

The metabolomics analysis of GXDK6 under 5–10% NaCl stress for 16 h indicated that 69 differential metabolites were performed (Figure 2C). These differential metabolites are mainly produced from the pathway of carbohydrate metabolism (e.g., D-fructose, D-mannose, 1-hexadecanol, and benzenepropanoic acid), amino acid metabolism (e.g., arginine, glutamate, and L-cysteine), and lipid metabolism (e.g., glycerol, methyl glycocholate, and tetrahydroxypentanoic acid-1,4-lactone). Moreover, several functional drug molecules such as ergotamine, deoxyspergualin, and calcitriol, were upregulated or downregulated under high NaCl stress, suggesting that the accumulation of these metabolites may contribute to regulating the salt tolerance survival of GXDK6.

**Figure 3:**
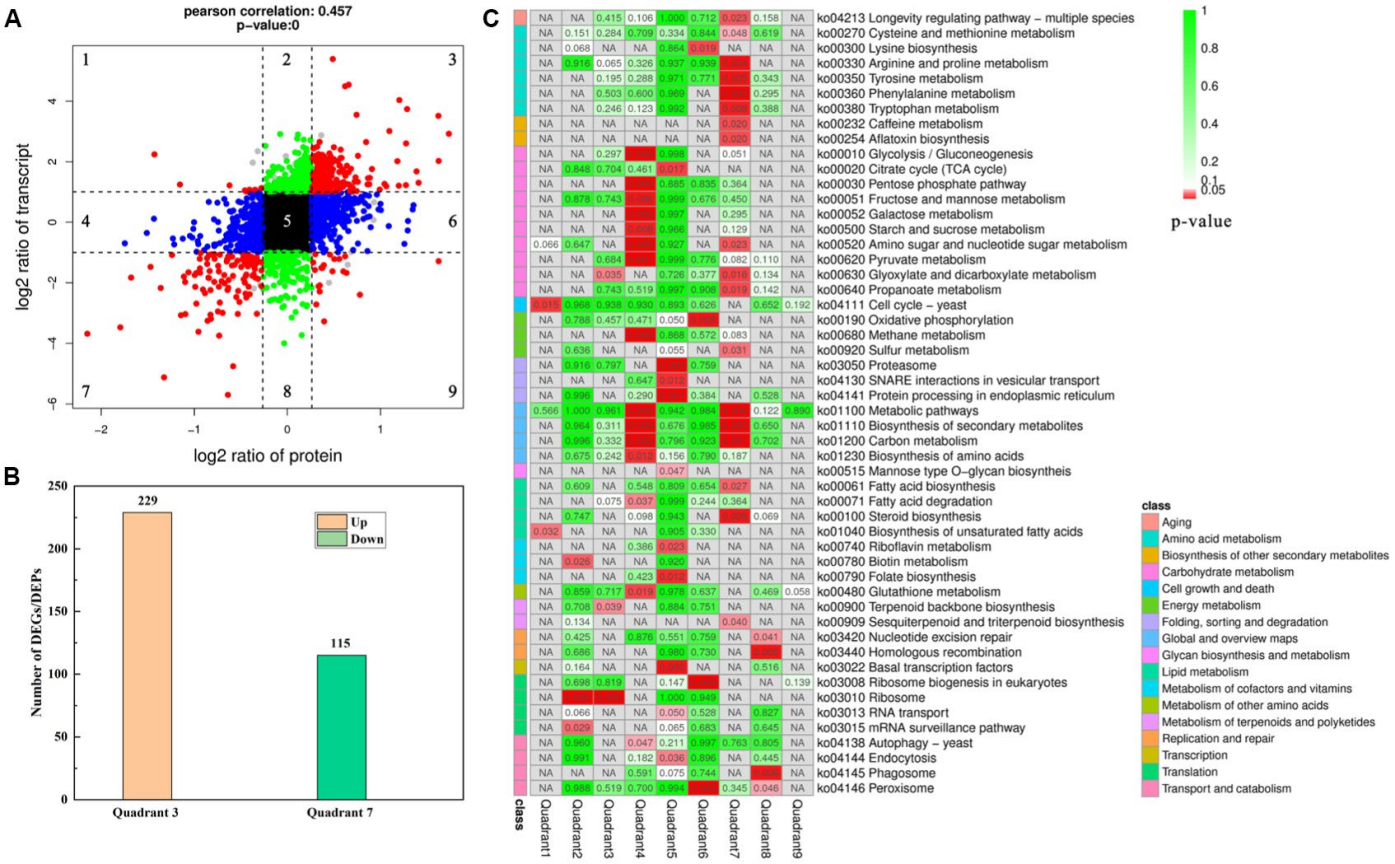
Association analysis of the omics data of GXDK6 under NaCl stress for 16 h. **(A)** Nine quadrants analyses of the associated genes/proteins in GXDK6; **(B)** Number of the DEGs and DEPs with consistent differential expression trend (upregulated or downregulated); **(C)** KEGG pathway analysis of the associated genes/proteins in GXDK6. In the panel **(A)**, the red color indicated that the genes and proteins were both significantly differentially expressed; green indicated the genes were differentially expressed but the proteins were not differentially expressed; blue indicated the proteins were differentially expressed but the genes were not differentially expressed; black indicated that neither genes nor proteins were significant differentially expressed. In the panel **(C)**, the different shades of red or green in the box indicated the value of *p* was low or high. When the associated DEGs/DEPs were significantly enriched in the pathways (*p* < 0.05), it was marked in red.

Association analysis of the omics data of GXDK6 indicated that all identified proteins (3,604, 100% of proteins detected) were associated with their corresponding genes. As shown in Figure 2B, 5,175 genes and 3,604 proteins were identified in GXDK6, each protein was matched with their corresponding gene, among which 344 DEGs and DEPs are coincident, indicating that these DEGs and DEPs showed the same differential expression trend (both were upregulated or downregulated). Furthermore, the total associated genes and proteins were divided into nine quadrants (Figure 3A). As shown in Figure 3A, the red color indicated that the genes and proteins were both differentially expressed. Green indicated the genes were differentially expressed but the proteins were not differentially expressed. Blue indicated the proteins were differentially expressed but the genes were not differentially expressed. Black indicated that neither genes nor proteins were differentially expressed. Among the nine quadrants, the first, second, and fourth quadrants indicated that the expression abundance of proteins is lower than that of mRNA, which indicates that they are post-transcriptional or translation-level regulation, for example, miRNA regulation of target gene inhibits the translation of related proteins. The third (upregulated) and seventh (downregulated) quadrants indicated the same trend of differential expression of mRNAs and proteins. The fifth quadrant indicated that genes and proteins have not differentially expressed. The sixth, eighth, and ninth quadrants indicated that the expression abundance of proteins was higher than that of mRNAs, suggesting that they were a process of post-transcriptional or translational level regulation or accumulation of proteins. Among the total associated DEGs/DEPs, 229 were up-regulated (Supplementary Table S7), whereas 115 were downregulated (Figure 3B; Supplementary Table S8). As the DEGs/DEPs located in the third or seventh quadrant indicated a consistent differential expression trend of genes and proteins, they were selected for the following analysis. Among the DEGs/DEPs located in the third or seventh quadrants, nine items, including aging, amino acid metabolism, biosynthesis of other secondary metabolites, carbohydrate metabolism, energy metabolism, global and overview maps, lipid metabolism, metabolism of terpenoids and polyketides, and translation, were identified (Figure 3C), suggesting that GXDK6 exhibits a complex survival regulatory network under high NaCl stress.

### Differentially expressed signal transduction proteins of GXDK6 under salt stress

According to the associated DEGs/DEPs with consistent differential expression trend, only one signal transduction protein (located in the third quadrant), which is phosphorelay intermediate protein YPD1, was upregulated under 10% NaCl stress. It controls the activity of the HOG1 pathway and gene expression in response to osmotic stress. Thus the upregulated phosphorelay intermediate protein YPD1 may activate the nuclear transcription factor activity (SKN7) and protein binding activity (SSK1) in the cytoplasm (Janiakspens et al., 2000), which could contribute to transmitting the salt stress signals to GXDK6 under high NaCl stress. Due to the signal conversion process, extremely salt-tolerant microorganisms usually endows themselves with different salt-tolerant viability (Menon et al., 2006; Ruas et al., 2019; Peng et al., 2021), the phosphorelay intermediate protein YPD1 from several typical fungi were compared, including *Saccharomyces cerevisiae, Candida albicans, Pichia kudriavzevii,* and *Komagataella phaffii*. The comparison results showed that the phosphorelay intermediate protein YPD1 in GXDK 6 contained only 147 amino acids, with a molecular weight of 16.88 kDa, and the conserved region was mainly located between amino acids 22–96th (Table 2). This protein has the highest homology with the phosphorelay intermediate protein YPD1 in *C. albicans* (similarity of 65.28%), whereas it had the lowest homology with *S. cerevisiae* (similarity of 37.59%). In *C. albicans*, the phosphorelay intermediate protein was known as the part of the bifurcated SLN1-YPD1-SKN7/SSK1 two-component regulatory system, which controls the activity of the HOG1 pathway and gene expression in response to oxidative stress, and probably the changes in the osmolarity of the extracellular environment (Kido et al., 1994). According to our omics data, the *YPD1* gene was upregulated by 2.53-fold under 10% NaCl stress, its corresponding protein was also upregulated by 1.65-fold. This suggests that the phosphorelay intermediate protein YPD1 in GXDK6 may be involved in the signal transduction process under salt stress, and is beneficial for GXDK6 to regulate the expression of its related genes and/or proteins, thereby contributing to the salt tolerance survival of GXDK6 (Donate-Correa et al., 2014).

**Table 2:** Amino acid sequence alignment of phosphorelay intermediate protein YPD1 in different fungi.

Coding gene	Species	Number of amino acids	MW (kDa)	Similarity of amino acid sequence
*YPD1*	*M. guilliermondii* GXDK6	147	16.88	100%
*YPD1*	*Saccharomyces cerevisiae*	167	19.17	37.59%
*YPD1*	*Candida albicans*	184	20.56	65.28%
*YPD1*	*Pichia kudriavzevii*	178	20.35	42.36%
*YPD1*	*Komagataella phaffii*	152	16.78	43.45%

These results were obtained by bioinformatics analysis using online websites including Uniprot, Expasy ProtParam tool (https://web.expasy.org/protparam/), and Clustal Omega (https://www.ebi.ac.uk/Tools/msa/clustalo/).

**Figure 4:**
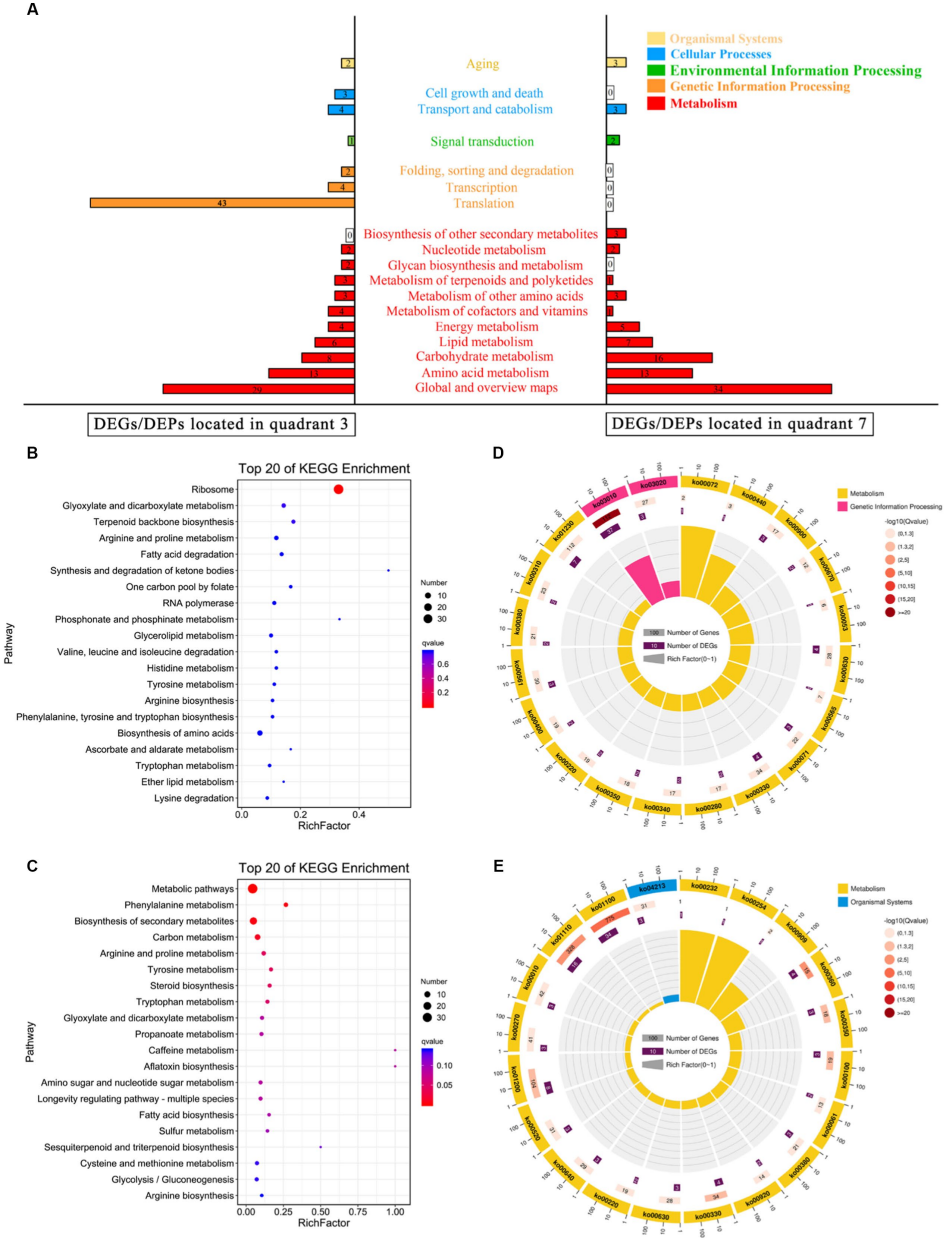
KEGG annotation analysis of the DEGs/DEPs. **(A)** KEGG enrichment of the DEGs/DEPs located in quadrant 3 or quadrant 7. **(B)** Top 20 KEGG enrichment of the upregulated DEGs/DEPs; **(C)** Top 20 pathway maps of the upregulated DEGs/DEPs; **(D)** Top 20 KEGG enrichment of the downregulated DEGs/DEPs; and **(E)** Top 20 pathway maps of the downregulated DEGs/DEPs.

However, two signal transduction-related DEGs (*GRP2* and *scaffold3.t827*) were identified (Figure 4A), and as their corresponding DEPs [NAD(P)-binding protein and Piso0_000808 protein] were significantly down-regulated under 10% NaCl stress. NAD(P)-binding protein is involved in regulating the putative NADPH-dependent methylglyoxal reductase GRP2 and catalyzing the irreversible reduction of the cytotoxic compound methylglyoxal (2-oxopropanal) to (S)-lactaldehyde, thereby contributing to the reduction of oxidative damage (Muramatsu et al., 2005). Piso0_000808 protein is relevant to carbohydrate metabolism. However, its detailed function has not been revealed yet. Therefore, these differentially expressed signal transduction proteins may be helpful to GXDK6 in regulating the differential expression of corresponding salt tolerance-related genes and proteins to resist high salt stress. Thus increasing the salt-resistant viability of GXDK6.

### DEGs/DEPs regulation pattern of GXDK6 under high salt stress

According to the multi-omics data, four DEGs (*SMD2, RPA43, RPA12, and RPB11*) and their corresponding DEPs (endoribonuclease YSH1, DNA-directed RNA polymerase I subunit RPA43, DNA-directed RNA polymerase I subunit RPA12, and DNA-directed RNA polymerase II subunit RPB11) were upregulated. These DEGs/DEPs were involved in regulating nucleic acid binding and RNA polymerase activity, and they may contribute to the regulation of the transcription level in GXDK6. In terms of translation, 43 upregulated DEGs (e.g., *MRPL1, RPL5,* and *RPS3*), and their corresponding DEPs (e.g., 54S ribosomal protein L1 mitochondrial, 60S ribosomal protein L5, and 40S ribosomal protein) were identified (Supplementary Table S9). They are involved in regulating purine ribonucleoside binding, aminoacyl-tRNA ligase activity, ion transmembrane transporter activity, and structural molecule activity, which may be conducive to supporting the salt tolerance survival of GXDK6 (Amunts et al., 2014; Pfeffer et al., 2015). Moreover, two DEGs (*RPN6,* and *OXA1*) and DEPs (26S proteasome regulatory subunit RPN6 and mitochondrial inner membrane protein OXA1) relevant to protein folding, sorting, and degradation were upregulated. They participated in regulating ribosome binding, ion transmembrane transporter activity, and the post-translational modification of protein (Isono et al., 2005), which could be helpful for the regulation of protein synthesis and processing in GXDK6.

### Involvement of DEGs/DEPs in cell growth and death, transport, catabolism, and aging

Regarding aging (Figure 4A), two upregulated DEGs (*MRP1* and *RSM26*)/DEPs (37S ribosomal protein MRP1 and 37S ribosomal protein S26) could improve the structural molecule activity and cation binding ability of GXDK6. Besides, three down-regulated DEGs (*HSP104, PNC1,* and *HST2*)/DEPs (heat shock protein, nicotinamidase, and NAD-dependent protein deacetylase HST2) contributed to inhibiting the NAD-dependent histone deacetylase activity, purine ribonucleoside binding ability, and anion binding ability (Coda et al., 2013). Accordingly, the energy release and the degree of intracellular histone deacetylation in GXDK6 could be downregulated (Tayel et al., 2013). These regulations may be beneficial in enhancing the anti-apoptotic ability of GXDK6 under high salt stress.

In terms of transport and catabolism in cellular processes (Figure 4A), four up-regulated DEGs (*MRP1, RSM26, FIS1,* and *SYM1*)/DEPs (37S ribosomal protein MRP1, 37S ribosomal protein S26, mitochondria fission 1 protein, and protein SYM1) were identified. They were relevant to regulating structural molecule activity, cellular response, and cation and tetrapyrrole binding capacities in GXDK6 (Trott and Morano, 2004). In addition, three DEGs (*fap2, CTT1,* and *ATG8*)/DEPs (fructosyl amino acid oxidase, catalase T, and autophagy-related protein 8) related to oxidoreductase activity and oxidative damage were down-regulated under high salt stress, which suggest low levels of oxidative stress in GXDK6 under these conditions. Moreover, among the cell growth and death, three up-regulated DEGs (*spg1, RAG1,* and *CKS1*)/DEPs (septum-promoting GTP-binding protein 1, hexose transporter 2, and cyclin-dependent kinases regulatory subunit), which are beneficial to regulate the nucleoside-triphosphatase activity, hexose transmembrane transporter activity, transferase activity, ubiquitin-like protein binding ability, and purine ribonucleoside binding ability in GXDK6 (Schmidt et al., 1997). These cellular processes play key role in response to salt stress.

### Metabolic regulation pattern of GXDK6 under salt stress

As shown in Figure 4A, most of the DEGs/DEPs are relevant to the metabolic regulation in GXDK6. With amino acid metabolism and carbohydrate metabolism as examples, the downregulated DEGs/DEPs (located in the seventh quadrant) related to amino acid metabolism were enriched in five pathways, namely, cysteine and methionine metabolism (Ko00270), arginine and proline metabolism (Ko00330), tyrosine metabolism (Ko00350), phenylalanine metabolism (Ko00360), and tryptophan metabolism (Ko00380) as shown in Figure 3C. According to this, 10% NaCl stress could inhibit the biosynthesis of L-cysteine, putrescine, 4-guanidino butanote, tyrosine, phenylpyruvate, phenylacetaldehyde, indoleacetate, and cinnavalininate in GXDK6, suggesting that these metabolites may not support the salt tolerance survival of GXDK6. However, the upregulated DEGs/DEPs relevant to amino acid metabolism (map00220, map00340, map00400, and map00410) suggested that the biosynthesis of glutamate, urea, ornithine, N4-acetyl-amino-butanoate, imidazole-4acetate, and β-alanine in GXDK6 were promoted under high NaCl stress. In summary, the part of amino acid metabolism in GXDK6 may be transformed to biosynthesize some metabolites (e.g., glutamate, urea, betaine, ornithine, and β-alanine) that could contribute to the enhancement of its salt tolerance survivability instead of producing metabolites that may not increase salt tolerance (e.g., L-cysteine, putrescine, 4-hydroxy-phenylacetaldehyde, and tyrosine).

In terms of carbohydrate metabolism, the upregulated DEGs/DEPs mainly enriched in the pathway of glyoxylate and dicarboxylate metabolism (Ko00630), suggested that the biosynthesis of acetoacetyl-CoA, citrate, and 3-phospho-D-glycerate in GXDK6 were promoted under 10% NaCl stress. However, the down-regulated DEGs/DEPs mainly enriched in the pathways including amino sugar and nucleotide sugar metabolism (Ko00520), and propanoate metabolism (Ko00640), then would contribute to the biosynthesis of chitoboise, fructose-6P, mannose-6P, malonyl-CoA, methylglyoxal, and L-lactaldehyde, respectively. The decomposition of hydrogen peroxide into O_2_, and the decomposition of formate into CO_2_ in GXDK6 could be hence reduced. These results suggested that the regulation of basic carbon metabolism in GXDK6 contributed to decreasing the oxidation reaction process, thereby preventing the biomembrane from peroxidative damage, and generating some functional metabolites that may be conductive to the enhancement of its anti-hypertonic ability.

In addition to amino acid metabolism and carbohydrate metabolism, the omics data indicated that the upregulated DEGs/DEPs may contribute to the promotion of ribosome processing, terpenoid backbone biosynthesis (e.g., geranyl PP, hexaprenyl PP, and farnesyl PP), and fatty acid degradation in GXDK6. The downregulated DEGs/DEPs could result in reduced sulfur metabolism, carbon metabolism, fatty acid biosynthesis, sesquiterpenoid, and triterpenoid biosynthesis in GXDK6. The top 20 significantly enriched pathways of the upregulated DEGs/DEPs are shown in Figure 4B, whereas the top 20 enriched metabolic pathways of the down-regulated DEGs/DEPs are shown in Figure 4C. However, five common pathways existed in both of the Figures, including glyoxylate and dicarboxylate metabolism, arginine and proline metabolism, tyrosine metabolism, arginine biosynthesis, and tryptophan metabolism. These genes/proteins, which were significantly upregulated or downregulated in the same pathway, suggested that the accumulation or reduction in some differential metabolites (e.g., β-alanine, urea, and L-cystein) contributed to regulating the salt tolerance survival of GXDK6. To further understand the top 20 KEGG enrichments of the DEGs/DEPs, specific KEGG pathways were screened in accordance with enrichment factor (which reflected the ratio between the number of DEGs belonging to this term and the number of all genes enriched in this term). As shown in Figure 4D, the upregulated DEGs/DEPs were significantly enriched in genetic information processing (ko03010 and ko03020) and metabolism (the top three pathways were ko0072, ko0440, and ko00900) in GXDK6. However, Figure 4E indicated that the down-regulated DEGs/DEPs were significantly enriched in the organismal systems (ko04213) and metabolism (the top three pathways were ko00232, ko00254, and ko00909). These findings suggested that GXDK6 could regulate the differential expression of its related genes/proteins/metabolites in response to high salt stress. In turn, it could contribute to driving and transforming the direction of its metabolic flow, reducing cellular oxidative damage, and promoting the biosynthesis and accumulation of some metabolites (e.g., urea and β-alanine), which could be beneficial for supporting the salt tolerance survival of GXDK6.

### Determination exogenous functional metabolites on viability of GXDK6 under salt stress

**Figure 5:**
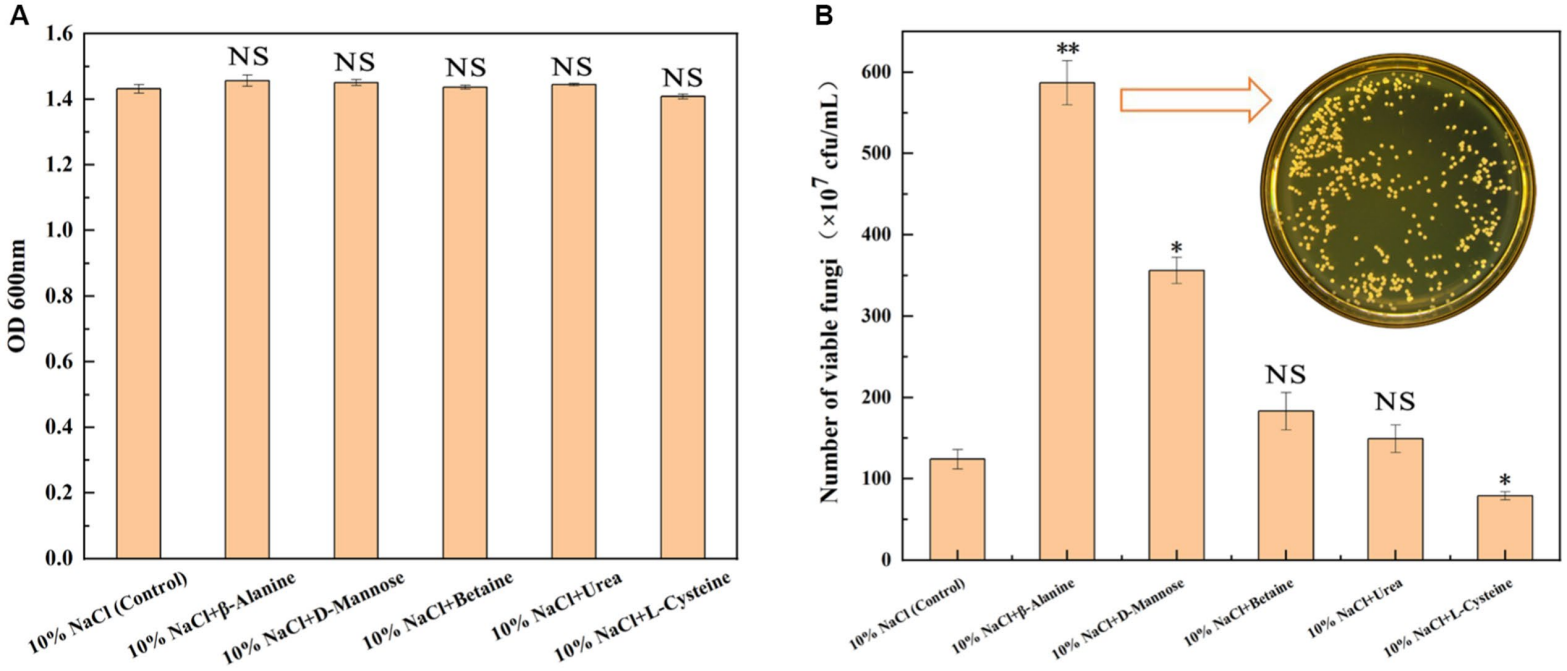
Effects of adding exogenous metabolites on the growth of GXDK6. **(A)** OD600 of GXDK6 incubated for 48 h under 10% NaCl stress; **(B)** Number of viable fungi of GXDK6 incubated for 48 h under 10% NaCl stress containing various metabolites (β-alanine, D-mannose, betaine, urea, and L-cysteine) with a final concentration at 100 mg/L. “^**^” indicated very significant difference compared with the control (*p* < 0.01); “^*^” indicated significant difference compared with the control (*p* < 0.05)”; and “NS” indicated not significant difference compared with the control (*p* > 0.05).

On the basis of the above multi-omics data analysis, five differential metabolites (β-alanine, D-mannose, urea, betaine, and L-cysteine) were screened to explore whether the exogenous addition of relevant metabolites could enhance the salt tolerance survival of GXDK6. As shown in Figure 5, the exogenous addition of 100 mg/L β-alanine, D-mannose, urea, and betaine enhanced the salt tolerance survivability of GXDK6 at varying degrees (Figure 5B) without affecting growth (Figure 5A). Among them β-alanine and D-mannose showed a significant promotion effect (Figure 5B, *p* < 0.05). For example, when GXDK6 was incubated for 48 h under 10% NaCl stress containing 100 mg/L of β-alanine, its viable fungi was 5.87 × 10^9^ cfu/mL, which was 3.85-fold higher than that of the control (1.21 × 10^9^ cfu/mL), indicating that β-alanine promoted the viability of GXDK6 significantly. However, the exogenous addition of 100 mg/L urea or betaine did not improve the salt tolerance survival of GXDK6 significantly (*p* > 0.05, Supplementary Figure S2). Moreover, L-cysteine was verified as the metabolite that could not support the salt tolerance survival of GXDK6. The viable fungi of GXDK6 incubated under 10% NaCl stress containing 100 mg/L of L-cysteine (7.9 × 10^8^ cfu/mL) was 35.54% lower than that of the control (1.21 × 10^9^ cfu/mL, Figure 5B). This result matched well with the finding that the biosynthesis of L-cysteine in GXDK6 could be inhibited under high NaCl stress.

### Validation of *YPD1* gene regulation of GXDK6 under salt stress

**Figure 6:**
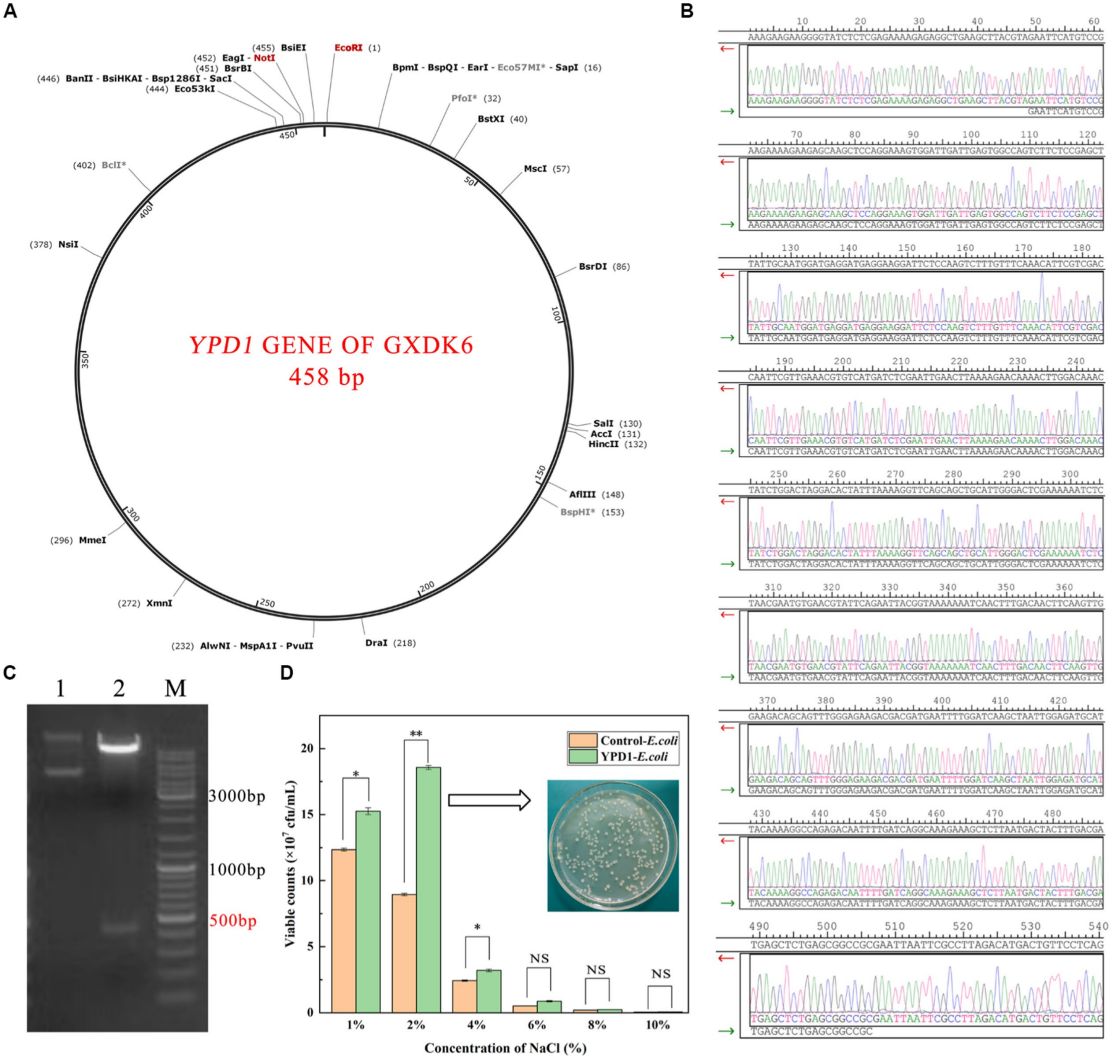
Cloning of *YPD1* gene from *Meyerozyma guilliermondii* GXDK6 in *E. coli.* **(A)** The length of *YPD1* gene of GXDK6; **(B)** Sanger sequencing results of the inserted sequence; **(C)** Agarose gel electrophoresis detection results of the *YPD1* gene (Lane 1: original plasmid, Lane 2: enzyme NotI/EcoRI and target plasmid, M: marker); **(D)** The viable counts of the *E.coli* and *YPD1-E.coli* incubated under 1–10% NaCl stress. “^**^” indicated very significant difference compared with the control (*p* < 0.01); “^*^” indicated significant difference compared with the control (*p* < 0.05)”; and “NS” indicated not significant difference compared with the control (*p* > 0.05).

As shown in Figure 6, the length of *YPD1* gene of GXDK6 is 458 bp, and the appropriate restriction site is EcoRI/NotI (Figure 6A). After the *YPD1* gene was inserted into *E.coli*, it was further verified by Sanger sequencing (Daniels et al., 2021) that the inserted gene sequence was consistent with the *YPD1* gene sequence of GXDK6 (Figure 6B), which indicated that the *YPD1* gene had been successfully cloned into *E.coli*, and the cloned strain *YPD1*-*E.coli* with kanamycin resistance was obtained. Furthermore, the results of agarose gel electrophoresis also verified that the electrophoretic band of *YPD1* gene in the cloned strain is between 450 and 500 ppb (Figure 6C), which met the expected target of 458 bp. Subsequently, the salt-tolerant viability of *YPD1*-*E.coli* within 48 h was tested by plate counting method. Results showed that the viable counts of *YPD1*-*E.coli* was remarkably higher than that of control-*E.coli* incubated under 1–6% NaCl (Figure 6D). For instance, the viable counts of *YPD1*-*E.coli* reached the highest value of 1.9 × 10^8^ cfu/mL when cultured under 2% NaCl, which was 113.48% higher than that of the control (8.9 × 10^7^ cfu/mL). In addition, even under 4 or 6% NaCl stress, the viable counts of *YPD1-Ecoli* reached 3.1 × 10^7^ and 9.2 × 10^6^ cfu/mL, which was 29.17 and 73.58% higher than that of the control (2.4 × 10^7^ and 5.3 × 10^6^ cfu/mL), respectively. However, when the concentration of NaCl increased to more than 8%, both of the *E.coli* and *YPD1-Ecoli* could hardly grow, suggesting that the maximum salt tolerance of them are both within 8% NaCl stress. These results indicated that *YPD1* gene of GXDK6 was an important salt-tolerant regulatory gene, which could obviously contribute to increasing the salt-tolerant viability of the strain.

## Discussion

*Meyerozyma guilliermondii* is a novel probiotic known for producing flavor compounds in fermented products, and it has been widely used in food fermentation in recent years. For example, *M. guilliermondii* was used to ferment wheat bread and synthesize many active compounds that could extend the shelf life of wheat bread effectively by 14 days (Jensen et al., 2009). Furthermore, *M. guilliermondii* was also known to be a typical extremely salt-tolerant microorganism, which could grow well and produce abundant functional metabolites under salt stress (≥10% NaCl; Mo et al., 2021). However, the salt stress regulation network of *M. guilliermondii* has not yet been clearly revealed. Most of the previous reports focused on the study of salt tolerance genes and proteins of *M. guilliermondii*, but paid little attention to its overall survival regulations. The understanding of its metabolic network is also lacking in systematic revelation (Akshya and Sukesh, 2017; Wang et al., 2021). In response to salt stress, extremely salt-tolerant microorganisms have evolved a variety of strategies. For example, Wang et al. (2021) revealed the importance of lipid metabolism and membrane permeability in regulating the morphological changes in cells. This regulation is due to the signal instructions of gene transcription and expression, which allow cells to avoid internal damage effectively in high-permeability environments. Therefore, the cell morphology of GXDK 6 sags and contracts under high NaCl stress, which may be helpful to reducing its specific surface area in contact with NaCl, and contribute to regulating its cell membranes permeability, thus supporting the survival of GXDK6 under high osmotic pressure. However, how the detailed regulatory signals work and whether such regulation will lead to significant changes in metabolic patterns still need further studies.

Salt-stress perception is the key to increase response rate, which may depend on the unique signal transduction process for extremely salt tolerance microorganisms (Peng et al., 2021). In this present work, an important upregulated signal transduction gene (*YPD1*) and its corresponding protein (phosphorelay intermediate protein YPD1) that was relevant to the salt-stress perception in GXDK6 were identified. This protein was compared with the currently known phosphorelay intermediate protein YPD1 in several typical fungi (including *Saccharomyces cerevisiae, Candida albicans, Pichia kudriavzevii,* and *Komagataella phaffii*). In *C. albicans*, the phosphorelay intermediate protein was known as the part of the bifurcated SLN1-YPD1-SKN7/SSK1 two-component regulatory system, which controls the activity of the HOG1 pathway and gene expression in response to oxidative stress, and probably the changes in the osmolarity of the extracellular environment (Kido et al., 1994). According to our omics data, the *YPD1* gene in GXDK6 was upregulated by 2.53-fold under 10% NaCl stress, and its corresponding protein was also upregulated by 1.65-fold. These findings suggested that the phosphorelay intermediate protein YPD1 in GXDK6 may be involved in the signal transduction process under salt stress, and beneficial for GXDK6 to regulate the expression of its related genes and proteins, thereby contributing to the salt tolerance survival of GXDK6 (Donate-Correa et al., 2014).

With regard to the survival mechanism of yeast under high salt stress, Lee et al. (2021) reported a large number of DEGs (up to 2,448 DEGs) in *Hyphopichia* yeast incubated under 1 M NaCl (equivalent to 5.8% NaCl) stress. Their transcriptome data were mapped to glycolysis pathway and sugar transporters, which revealed two processes on how *Hyphopichia* yeasts increased their intracellular concentration of glycerol: (i) enhanced glycerol production via the upregulation of two key glycerol biosynthesis genes, namely *GPD1* and *GPP2*; and (ii) increased glycerol uptake via upregulation of *STL1*, encoding a glycerol/H+ symporter in the plasma membrane. These genes were highly induced by NaCl stress. Furthermore, their transcriptome data revealed the downregulation of most *ERG* genes involved in the ergosterol biosynthesis pathway in *Hyphopichia*, which implies decreased levels of ergosterol in the plasma membrane upon encountering salt stress. The authors demonstrated that these changes are important to *Hyphopichia* for stress resistance. Our transcriptome data also identified some related DEGs (e.g., the up-regulated *GPP1, FPS1,* and *GUT1*). Moreover, we confirmed that the corresponding proteins of these genes were up- or downregulated by proteomic sequencing analysis, and these DEGs/DEPs were involved in transforming the metabolic network of GXDK6, thereby producing some differential metabolites that maybe contribute to the viability of GXDK6 under NaCl stress.

In addition, the metabolic regulation network in GXDK6 had been elucidated systematically using integrative omics technology (genome, transcriptome, proteome, and metabolome) in this work. The results showed that up to 65.9% of the total associated DEGs/DEPs (980 types) were relevant to the metabolic regulation in GXDK6 (e.g., amino acid metabolism, carbohydrate metabolism, and lipid metabolism), and these DEGs/DEPs contributed to producing many functional metabolites (e.g., β-alanine and D-mannose) and drug molecules (e.g., deoxyspergualin and calcitriol). Among drug molecules, deoxyspergualin was an important immunosuppressant suitable for acute rejection after kidney transplantation; it has been widely used in the clinical treatment of rejection after surgical transplantation (Kirk et al., 2005). Calcitriol is a non-selective vitamin D receptor activator, that could be used to treat renal dystrophy, hypoparathyroidism, and vitamin D-dependent and -resistant rickets in patients with chronic renal failure (Gutierrez, 2005). However, few studies showed that deoxyspergualin or calcitriol could be biosynthesized in *M. guilliermondii* under salt stress. Our findings will contribute to the development and biosynthesis of new molecular drugs using extremely salt tolerance microorganisms.

In terms of how to further improve the salt tolerance of microorganisms, several studies tended to modify specific genes artificially or express salt tolerance-related proteins to create new extremely salt tolerance microorganisms (by strategies such as gene knockout, gene cloning or gene overexpression). These methods undoubtedly received many positive results (Iwaki et al., 2010). However, the transformation of genetic elements belongs to the artificial construction of genetically engineered bacteria. For probiotics, the biosafety after transformation is worrying. People usually tend to use natural probiotics without any gene modification. Therefore, the exogenous addition of some conventional metabolites showed a greater advantage in improving the survival and metabolic capacities of salt-tolerant microorganisms. On the basis of the multi-omics data analysis results, five differential metabolites (D-mannose, L-cysteine, urea, betaine, and β-alanine) in GXDK6 were screened. The biosynthesis and accumulation of β-alanine or D-mannose were validated further to contribute positively to the salt tolerance survival of GXDK6. These metabolites were defined as signature metabolites that could promote the salt tolerance survival of GXDK6, thereby providing new insights into the subsequent studies on the survival regulation network of extremely salt tolerance microorganisms.

## Conclusion

In summary, a marine extreme salt-tolerant *M. guilliermondii* GXDK6, which could survive well under high NaCl stress was investigated. The findings confirmed that GXDK6 contained many genes, which could contribute to increasing its salt tolerant survival. In addition, the stress perception and metabolic network in GXDK6 were investigated further on the basis of integrated omics study. Many DEGs/DEPs in GXDK6 were found relevant to the regulation of genetic information processing, cellular processes, organismal systems, environmental information processing, and metabolism under high NaCl stress. Some of the metabolites (e.g., β-alanine and D-mannose) were confirmed as functional metabolites that could increase the viability of GXDK6 under high salt stress. In addition, NaCl stress could stimulate GXDK6 to biosynthesize many important drug molecules (e.g., deoxyspergualin and calcitriol). The present study contributed to further understanding the salt tolerance survival of *M. guilliermondii*, and it could be used for exploring novel functional products and/or drugs from extremely salt tolerance microorganisms.

## Data availability statement

The datasets presented in this study can be found in online repositories. The names of the repository/repositories and accession number(s) can be found at: the Whole Genome Shotgun project have been deposited in DDBJ/ENA/GenBank with the accession JAIGNZ000000000. The RNA-Sequencing data of GXDK6 under NaCl stress have been deposited in GenBank with the accession number PRJNA752222.

## Author contributions

CJ and XC conceived the study. XC, HS, and HB performed the experiments. XC wrote the manuscript. PS, BY, and XZ revised the manuscript. XC, HS, and HB analyzed the experimental data. All authors contributed to the article and approved the submitted version.

## Funding

This work was supported by the Funding Project of Chinese Central Government Guiding to the Guangxi Local Science and Technology Development (Grant No. GUIKEZY21195021), Natural Science Fund for Distinguished Young Scholars of Guangxi Zhuang Autonomous Region of China (Grant No. 2019GXNSFFA245011), the Innovation Project of Guangxi Graduate Education (Grant No. YCBZ2021012), and the Science Research and Technology Development Project of Jiangnan District of Nanning (Grant no. 20220620-5).

## Conflict of interest

The authors declare that the research was conducted in the absence of any commercial or financial relationships that could be construed as a potential conflict of interest.

## Publisher’s note

All claims expressed in this article are solely those of the authors and do not necessarily represent those of their affiliated organizations, or those of the publisher, the editors and the reviewers. Any product that may be evaluated in this article, or claim that may be made by its manufacturer, is not guaranteed or endorsed by the publisher.

## Supplementary material

The Supplementary material for this article can be found online at: https://www.frontiersin.org/articles/10.3389/fmicb.2023.1193352/full#supplementary-material

## Footnotes

^1^http://nmdc.cn
