# Supplementary material for: Salt stress perception and metabolic regulation network analysis of a marine probiotic Meyerozyma guilliermondii GXDK6
Source: Front Microbiol. 2023 Jul 17;14:1193352. doi: 10.3389/fmicb.2023.1193352 (PMC10387536; doi:10.3389/fmicb.2023.1193352)

**Figure S2. Effects of adding exogenous metabolites on the growth of GXDK6. (a)** Viable fungi of GXDK6 incubated under 10% NaCl stress. (**b**) Viable fungi of GXDK6 incubated under 10% NaCl stress containing 100 mg/L β-alanine; (**c**) Viable fungi of GXDK6 incubated under 10% NaCl stress containing 100 mg/L D-mannose; (**d**) Viable fungi of GXDK6 incubated under 10% NaCl stress 100 mg/L betaine; (**e**) Viable fungi of GXDK6 incubated under 10% NaCl stress containing 100 mg/L urea; (**f**) Viable fungi of GXDK6 incubated under 10% NaCl stress containing 100 mg/L L-cysteine.


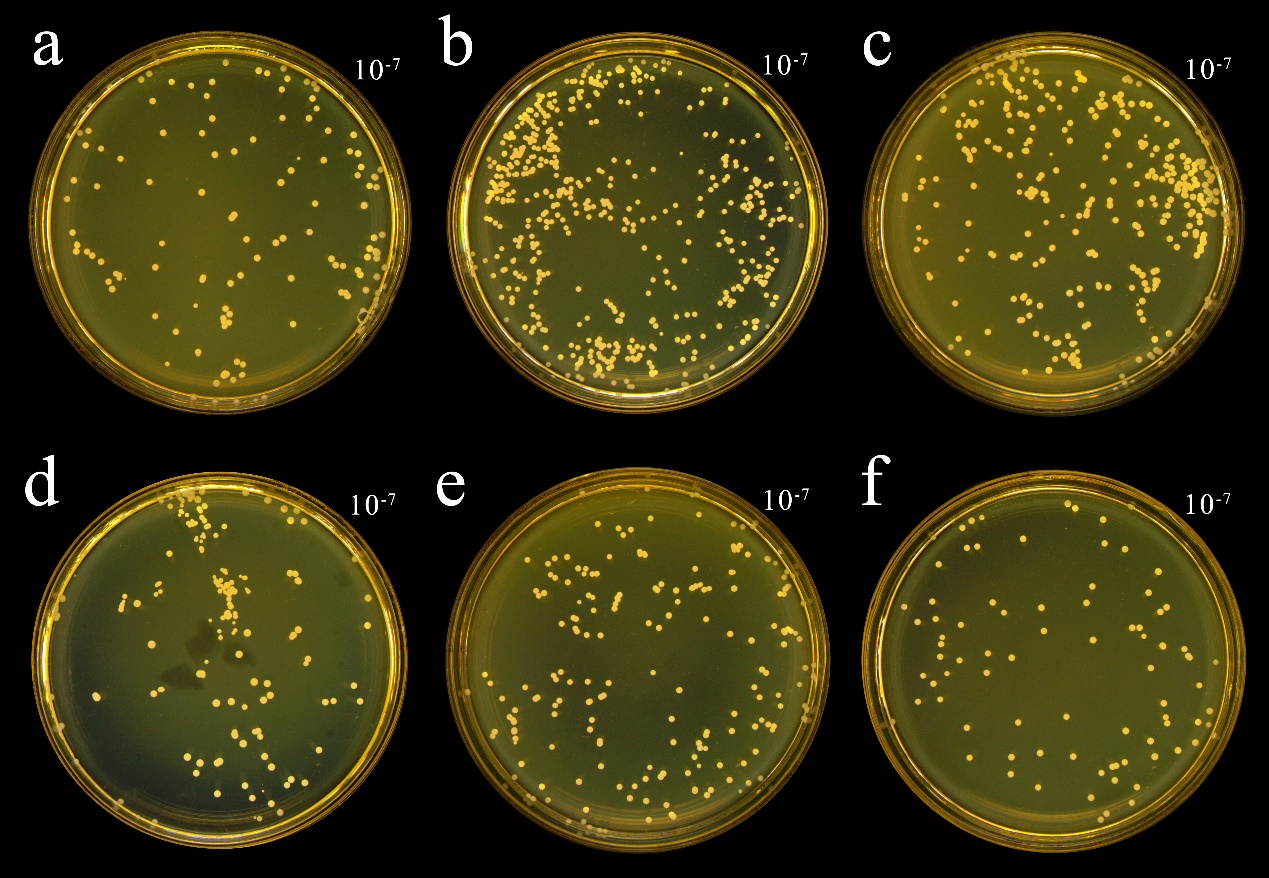

Supplement: Supplementary file 1 [file Data_Sheet_1.zip › Figure S2.DOCX]
