# Supplementary figures and images for: Salt stress perception and metabolic regulation network analysis of a marine probiotic Meyerozyma guilliermondii GXDK6
Source: Front Microbiol. 2023 Jul 17;14:1193352. doi: 10.3389/fmicb.2023.1193352 (PMC10387536; doi:10.3389/fmicb.2023.1193352)

**Figure S1. RT-qPCR verification of the selected DEGs of GXDK6.**

**
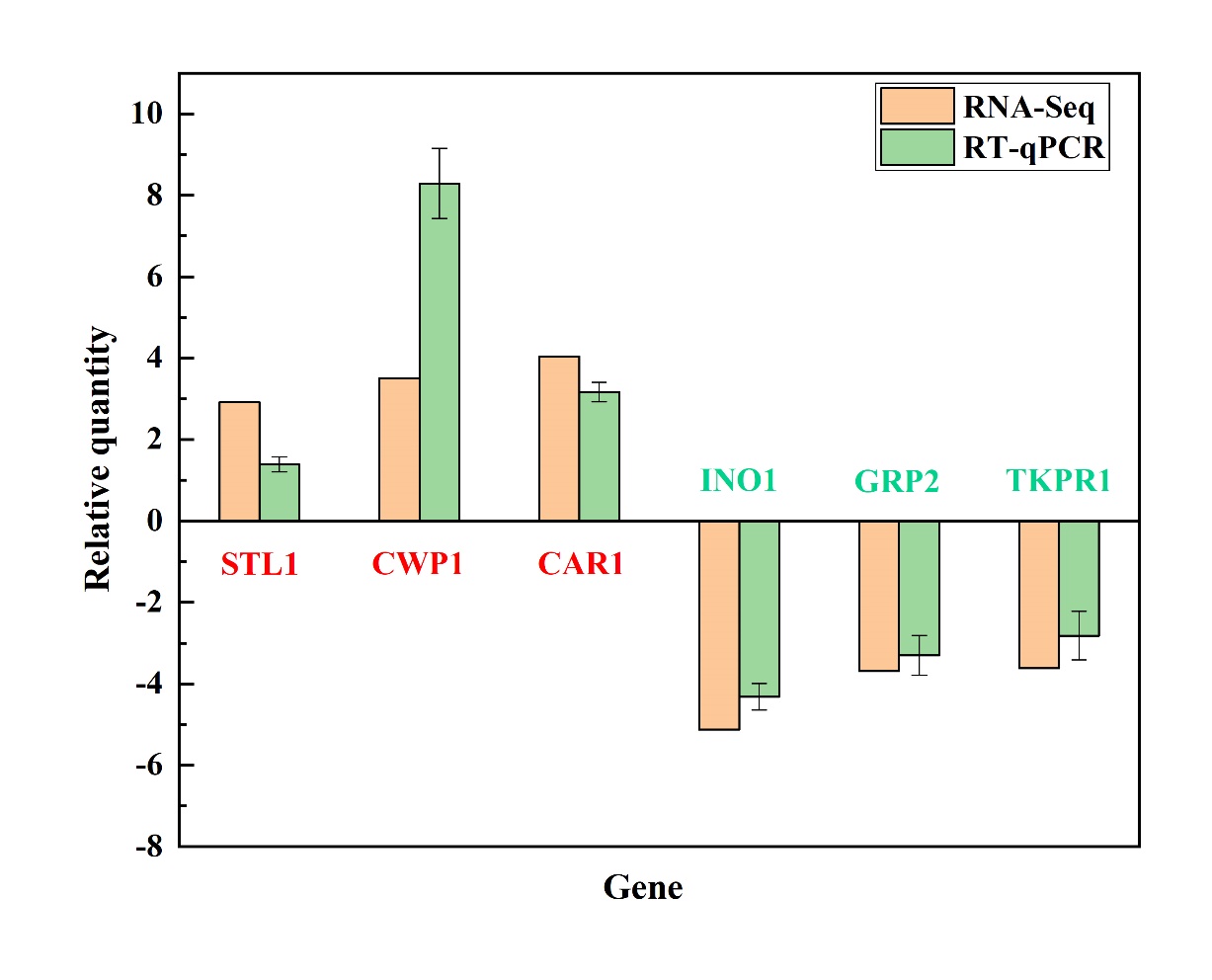
**

Supplement: Supplementary file 1 [file Data_Sheet_1.zip › Figure S1.DOCX]
